# Bone morphogenetic protein-2 functions as a negative regulator in the differentiation of myoblasts, but not as an inducer for the formations of cartilage and bone in mouse embryonic tongue

**DOI:** 10.1186/1471-213X-11-44

**Published:** 2011-07-07

**Authors:** Kayoko Aoyama, Akira Yamane, Takeo Suga, Erika Suzuki, Tadayoshi Fukui, Yoshiki Nakamura

**Affiliations:** 1 Department of Orthodontics, Tsurumi University School of Dental Medicine, Yokohama, Japan; 2 Department of Biophysics, Tsurumi University School of Dental Medicine, Yokohama, Japan; 3 Department of Geriatric Dentistry, Tsurumi University School of Dental Medicine, Yokohama, Japan

## Abstract

**Background:**

In vitro studies using the myogenic cell line C2C12 demonstrate that bone morphogenetic protein-2 (BMP-2) converts the developmental pathway of C2C12 from a myogenic cell lineage to an osteoblastic cell lineage. Further, in vivo studies using null mutation mice demonstrate that BMPs inhibit the specification of the developmental fate of myogenic progenitor cells. However, the roles of BMPs in the phases of differentiation and maturation in skeletal muscles have yet to be determined. The present study attempts to define the function of BMP-2 in the final stage of differentiation of mouse tongue myoblast.

**Results:**

Recombinant BMP-2 inhibited the expressions of markers for the differentiation of skeletal muscle cells, such as myogenin, muscle creatine kinase (MCK), and fast myosin heavy chain (fMyHC), whereas BMP-2 siRNA stimulated such markers. Neither the recombinant BMP-2 nor BMP-2 siRNA altered the expressions of markers for the formation of cartilage and bone, such as osteocalcin, alkaline phosphatase (ALP), collagen II, and collagen X. Further, no formation of cartilage and bone was observed in the recombinant BMP-2-treated tongues based on Alizarin red and Alcian blue stainings. Neither recombinant BMP-2 nor BMP-2 siRNA affected the expression of inhibitor of DNA binding/differentiation 1 (Id1). The ratios of chondrogenic and osteogenic markers relative to glyceraldehyde-3-phosphate dehydrogenase (GAPDH, a house keeping gene) were approximately 1000-fold lower than those of myogenic markers in the cultured tongue.

**Conclusions:**

BMP-2 functions as a negative regulator for the final differentiation of tongue myoblasts, but not as an inducer for the formation of cartilage and bone in cultured tongue, probably because the genes related to myogenesis are in an activation mode, while the genes related to chondrogenesis and osteogenesis are in a silencing mode.

## Background

The development of skeletal muscle proceeds through five phases, as follows [1]: phase 1 (specification), muscle progenitor cells are specified to become muscle cells in somites; phase 2 (migration), the muscle progenitor cells migrate to the presumptive places where muscles are formed; phase 3 (proliferation), the muscle progenitor cells proliferate, increase in number, and become myoblasts; phase 4 (differentiation), the myoblasts fuse to become multinucleated myotubes; phase 5 (maturation), the multinucleated myotubes mature to myofibers, such as fast-twitch myofibers or slow-twitch myofibers.

Bone morphogenetic proteins (BMPs) are members of the transforming growth factor β (TGFβ) super family and comprise a highly conserved and expanding family of 15 genes. BMPs were discovered as a factor that induced the ectopic formation of cartilage and bone when implanted intramuscularly in adult rats [2,3]. Since then, they have been found to play roles in many biological functions [4-6] including in the development of skeletal muscle.

In vitro studies using the myogenic cell line C2C12 demonstrate that BMP-2 converts the developmental pathway of C2C12 from a myogenic lineage to an osteoblastic lineage by reducing the activity of the myoD family, such as that of myoD and myogenin, and by up-regulating inhibitor of DNA binding/differentiation 1 (Id1) and runt-related gene 2 (Runx2) [7-11]. This conversion of the developmental pathway of C2C12 seems to inhibit myogenic differentiation, including myotube formation. In vivo studies using null mutation mice demonstrate that BMPs inhibit the specification of the developmental fate of myogenic progenitor cells: BMPs from the lateral plate and dorsal neural tube inhibit the specification in the somites and somitomeres [12-17], and Noggin (a BMP antagonist) suppresses the action of BMPs [18-20]. We recently reported that BMPs and their receptors are expressed in the myoblasts and myotubes of mouse embryonic tongues which are actively differentiating and maturating, implying that BMPs play a role in myoblast differentiation [21].

The tongue is a complex muscular organ comprised of several intrinsic and extrinsic muscles, and is involved in several important physiological tasks, such as suckling, swallowing, mastication, respiration, and vocalization. The tongue muscles constitute a subset of the head muscles, but several lines of evidence indicate that the program governing tongue myogenesis is more similar to those for migratory hypaxial muscles, such as the limb and diaphragm muscles, than to the head muscles, such as the masseter and temporalis muscles [22,23]. We have extensively studied the roles of peptide growth factors such as insulin-like growth factor (IGF) [24] and hepatocyte growth factor (HGF) in the development of tongue muscle cells using an organ culture system of mouse embryonic tongue [25,26]. The organ culture system of mouse embryonic tongue seems to be a good model for studying the genetic program governing migratory hypaxial myogenesis and for relating the results of in vivo studies with those of in vitro studies using established myogenic cell lines such as C2C12. However, the roles of BMPs in the phases of differentiation and maturation in skeletal muscles have yet to be fully elucidated. The present study attempts to define the functions of BMP-2 in the differentiation phase of myoblasts in mouse embryonic tongue using an organ culture system of embryonic day (E) 13 mouse tongue in which the differentiation phase of myoblasts is initiated [23].

## Results

### Recombinant BMP-2 had neither inhibitory nor proliferative effects on cultured tongue

**Figure 1:**
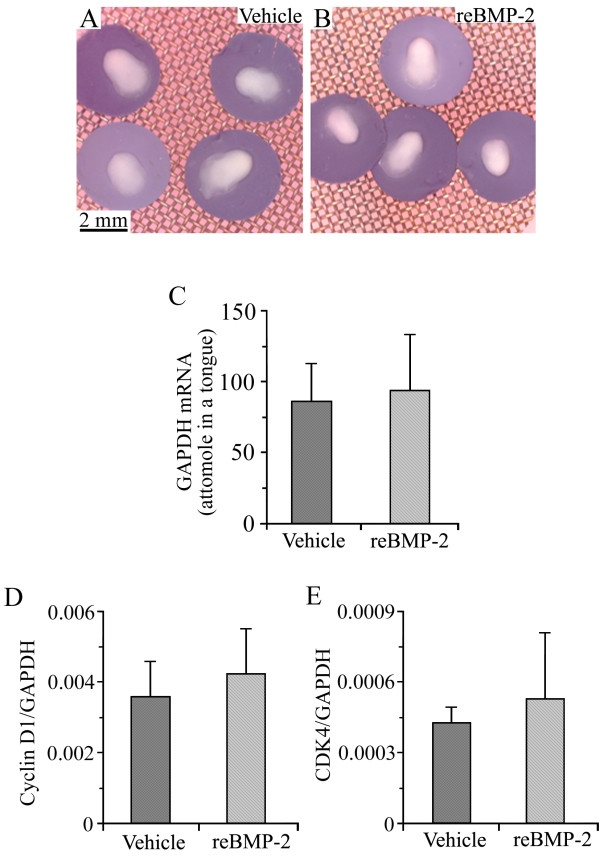
**Recombinant BMP-2 had neither inhibitory nor proliferative effects on cultured tongue**. Gross morphology of E13 tongues cultured for 8 days in BGJb medium containing vehicle (A) and 4 μg/ml of human recombinant BMP-2 (B). B used the same magnification as A. Expression level of GAPDH mRNA in E13 tongues cultured for 8 days in BGJb medium containing vehicle and 4 μg/ml of human recombinant BMP-2 (C). The longitudinal axis in C represents the quantity of GAPDH mRNA contained in one cultured tongue. Expression levels of cyclin D1 (D) and CDK4 (E) mRNAs in E13 tongues cultured for 8 days in BGJb medium containing vehicle and 4 μg/ml of human recombinant BMP-2. The longitudinal axis in D and E represents the ratio of target gene mRNA relative to GAPDH mRNA. Each column and vertical bar represent the mean + 1SD of six cultured tongues.

To identify possible toxic effects of recombinant BMP-2 on cultured tongue, we observed the gross morphology of E13 tongues cultured for 8 days in BGJb medium containing vehicle (Figure 1A) or recombinant BMP-2 (Figure 1B). No significant difference in the shape or size was observed between the vehicle- and recombinant BMP-2-treated tongue. To estimate the whole tissue volume of the cultured tongues, we measured the expression level of the mRNA of glyceraldehyde-3-phosphate dehydrogenase (GAPDH), a house keeping gene (Figure 1C). No marked difference was found between the expression level of GAPDH mRNA stimulated by the vehicle and that stimulated by recombinant BMP-2, suggesting that the recombinant BMP-2 had no inhibitory effect on the cultured tongues. Furthermore, we analyzed the mRNA expression levels of cyclin D1 (Figure 1D) and cyclin-dependent kinase 4 (CDK4) (Figure 1E), markers for the proliferation of cells, and found no significant difference between the vehicle and recombinant BMP-2, suggesting that recombinant BMP-2 did not alter the proliferation of cells in the cultured tongues.

### Recombinant BMP-2 suppressed the differentiation of myoblasts in cultured tongue

To identify the role of BMP-2 in the differentiation of tongue myoblasts, we supplemented human recombinant BMP-2 in the culture medium and analyzed the mRNA expression levels of myoD, myogenin, and muscle creatine kinase (MCK), markers for muscle differentiation, in the cultured tongue (Table 1 and Additional file 1). The ratios of myogenin and MCK, a marker for the final stages of muscle differentiation, relative to GAPDH in the recombinant BMP-2-treated tongues were 0.0180 ± 0.00162 and 0.0414 ± 0.00737, respectively, which were approximately 30 and 38% lower than those in the vehicle-treated tongues (0.0256 ± 0.00510 and 0.0661 ± 0.0149, respectively; p < 0.01). No significant difference in the expression level of myoD was found between the samples treated with the vehicle and those treated with recombinant BMP-2.

**Table 1:** mRNA levels for genes related to myogenesis, chondrogenesis, and osteogenesis in vehicle- and BMP-2-treated tongues

Genes	Vehicle	Recombinant BMP-2	Significance
Myogenesis			
MyoD	0.0163 ± 0.00616	0.0172 ± 0.00467	NS
Myogenin	0.0256 ± 0.00510	0.0180 ± 0.00162	p < 0.01
MCK	0.0661 ± 0.0149	0.0414 ± 0.00737	p < 0.01
Chondrogenesis, osteogenesis			
Runx2	0	0.0000661 ± 0.0000800	NS
Osteocalcin	0.0000514 ± 0.0000552	0.0000581 ± 0.0000535	NS
ALP	0.000813 ± 0.000632	0.000700 ± 0.000576	NS
Collagen II	0.000167 ± 0.00246	0.000535 ± 0.0105	NS
Collagen X	0.000129 ± 0.00123	0.000155 ± 0.00168	NS

The mean ± standard deviation of six vehicle-treated or six BMP-2-treated tongues for each gene is shown. The ratio of each target gene mRNA relative to GAPDH mRNA is shown.

**Figure 2:**
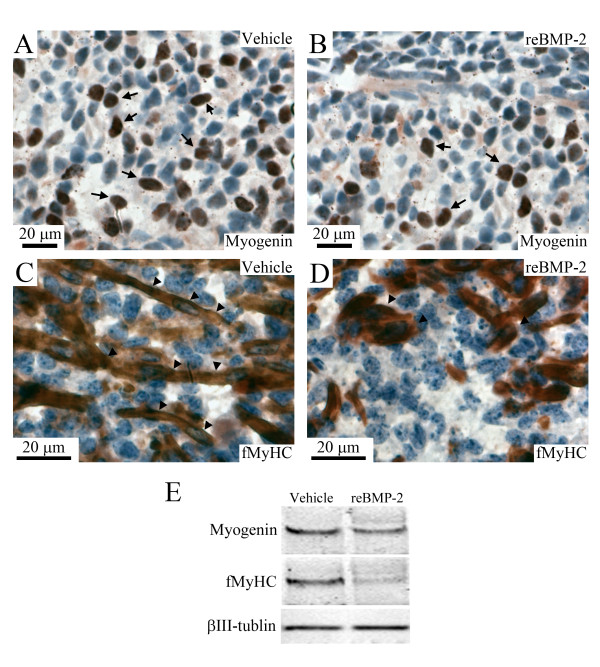
**Recombinant BMP-2 suppressed the expressions of myogenin and fMyHC in cultured tongue**. Immunostaining image for myogenin (A, B) and fMyHC (C, D) in the middle portion of E13 tongues cultured for 8 days in BGJb medium containing the vehicle (A, C) and 4 μg/ml of recombinant BMP-2 (B, D). Brown color indicates immunostaining image for myogenin and fMyHC. Arrows in A and B indicate myogenin-positive nuclei and arrowheads in C and D indicate elongated myotubes and myofibers. Western blotting pattern of myogenin, fMyHC, and βIII-tubulin in the whole portion of E13 tongues cultured for 8 days in BGJb medium containing the vehicle and 4 μg/ml of recombinant BMP-2 (E).

To further verify whether the recombinant BMP-2 suppressed the differentiation of tongue myoblasts in cultured tongues, we analyzed the localization and expression level of myogenin and fast myosin heavy chain (fMyHC) proteins in the recombinant BMP-2- or vehicle-treated tongues by immunohistochemical and Western blotting analyses (Figure 2). The number of myogenin-positive cells appeared to be lower in the BMP-2-treated tongue (arrows in Figure 2B) than in the vehicle-treated tongue (arrows in Figure 2A). Elongated myotubes with multi-nuclei were nearly absent in the BMP-2-treated tongues (arrowheads in Figure 2D), whereas they were clearly present in the vehicle-treated tongue (arrowheads in Figure 2C). The expression levels of myogenin and fMyHC proteins were lower in the BMP-2-treated tongue than in the vehicle-treated tongue (Figure 2E).

### Recombinant BMP-2 did not induce the formation of cartilage and bone in cultured tongue

To determine whether recombinant BMP-2 induced the formation of cartilage and bone in the cultured tongue, we analyzed the mRNA expression levels of Runx2, alkaline phosphatase (ALP), collagen II, and collagen X, which are markers for chondrogenesis and osteogenesis (Table 1 and Additional file 1). In the BMP-2-treated tongue, Runx2 mRNA was induced, but the ratio of Runx2 relative to GAPDH was extremely low and approximately 1000-fold lower than those of myogenic markers such as myoD, myogenin, and MCK. There was no significant difference in the mRNA expression levels of osteocalcin, ALP, collagen II, and collagen X between the recombinant BMP-2 and vehicle-treated tongues. Their ratios relative to GAPDH were also extremely low, being approximately 1000-fold lower than those of the myogenic markers, and the variations for measurement were also quite large.

**Figure 3:**
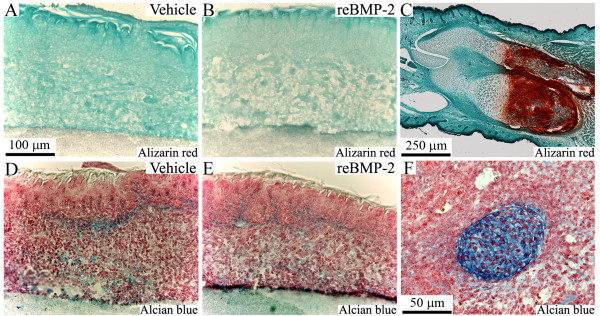
**Recombinant BMP-2 did not induce the formation of cartilage and bone in cultured tongue**. Staining images with Alizarin red (A, B, C) and Alcian blue (D, E, F) in the middle portion of E13 tongues cultured for 8 days in BGJb medium containing the vehicle (A, D) and 4 μg/ml of recombinant BMP-2 (B, E). Staining images of longitudinal section of hind limb containing tibia and fibula of E17 embryo and Meckel's cartilage of E11 embryo are shown as a positive control of Alizarin red and Alcian blue in C and F, respectively. B, D, and E used the same magnification as A.

To further determine whether recombinant BMP-2 induced the formation of cartilage and bone in the cultured tongue, we stained the cultured tongue using Alizarin red (Figure 3A, B, C) and Alcian blue (Figure 3D, E, F). No Alizarin red-positive staining was observed in any sample, but a weak, nearly identical Alcian blue-positive staining was observed in both the BMP-2- and vehicle-treated tongues.

### Suppression of BMP-2 expression by siRNA had neither inhibitory nor proliferative effects on cultured tongue

**Figure 4:**
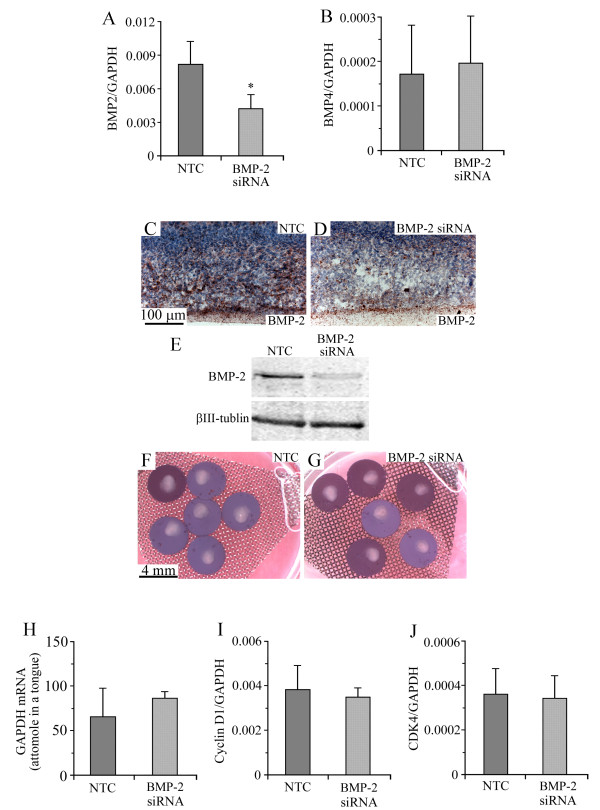
**BMP-2 siRNA suppressed the expression of BMP-2, but had neither inhibitory nor proliferative effects**. Expression levels of BMP-2 (A) and BMP-4 (B) mRNAs in E13 tongues cultured for 8 days in BGJb medium containing 250 nM of NTC and BMP-2 siRNA. Immunostaining image for BMP-2 in the middle portion of E13 tongues cultured for 8 days in BGJb medium containing NTC (C) and BMP-2 siRNA (D). Western blotting pattern of BMP-2 and βIII-tubulin in the whole portion of E13 tongues cultured for 8 days in BGJb medium containing the NTC and BMP-2 siRNA (E). Gross morphology of E13 tongues cultured for 8 days in BGJb medium containing NTC (F) and BMP-2 siRNA (G). Expression level of GAPDH mRNA in cultured E13 tongues (H). The longitudinal axis in H represents the quantity of GAPDH mRNA contained in one cultured tongue. Expression levels of cyclin D1 (I) and CDK4 (J) mRNAs in cultured E13 tongues. The longitudinal axis in I and J represents the ratio of target gene mRNA relative to GAPDH mRNA. Each column and vertical bar represent the mean + 1SD of six cultured tongues.

The addition of BMP-2 siRNA to the cultured medium induced an approximately 50% suppression of the expression of BMP-2 mRNA (Figure 4A), but no suppression in BMP-4 mRNA (Figure 4B). The result of immunohistochemical and Western blotting analyses indicated that BMP-2 siRNA induced a marked suppression in the expression of BMP-2 protein compared with non-target control RNA (NTC) (Figure 4C, D, E). The suppression of BMP-2 by siRNA induced no significant difference in the gross morphology of the cultured tongue (Figure 4F, G). Neither was there a significant difference in the expressions of GAPDH (Figure 4H), cyclin D1 (Figure 4I), and CDK4 (Figure 4J) between the samples treated with NTC and those treated with BMP-2 siRNA, suggesting that BMP-2 siRNA had neither inhibitory nor proliferative effects on cultured tongue.

### BMP-2 siRNA stimulated the differentiation of myoblasts in cultured tongue

To determine the role of BMP-2 in the differentiation of tongue myoblasts, we analyzed the effect of BMP-2 siRNA on the mRNA expression levels of myoD, myogenin, and MCK in cultured tongue (Table 2 and Additional file 1). The ratios of myogenin and MCK relative to GAPDH in the BMP-2 siRNA-treated tongues were 0.0203 ± 0.0037 and 0.116 ± 0.0275, respectively, which were approximately 60 and 80% higher than those in the NTC-treated tongues (0.0128 ± 0.0044 and 0.0646 ± 0.0251, respectively; p < 0.01). No significant difference in the expression levels of myoD was found between the NTC- and BMP-2 siRNA-treated cultures.

**Table 2:** mRNA levels for genes related myogenesis, chondrogenesis, and osteogenesis in NTC- and BMP-2 siRNA-treated tongues

Genes	NTC	BMP-2 siRNA	Significance
Myogenesis			
MyoD	0.0130 ± 0.00452	0.0136 ± 0.00515	NS
Myogenin	0.0128 ± 0.0044	0.0203 ± 0.0037	p < 0.01
MCK	0.0646 ± 0.0251	0.116 ± 0.0275	p < 0.01
Chondrogenesis, osteogenesis			
Runx2	0	0	NS
Osteocalcin	0.0000406 ± 0.0000632	0.0000356 ± 0.0000795	NS
ALP	0.00291 ± 0.00455	0.00365 ± 0.00460	NS
Collagen II	0.000202 ± 0.000333	0.0000435 ± 0.0000529	NS
Collagen X	0.000674 ± 0.000690	0.000264 ± 0.000265	NS

The mean ± standard deviation of six vehicle-treated or six BMP-2-treated tongues for each gene is shown. The ratio of each target gene mRNA relative to GAPDH mRNA is shown.

**Figure 5:**
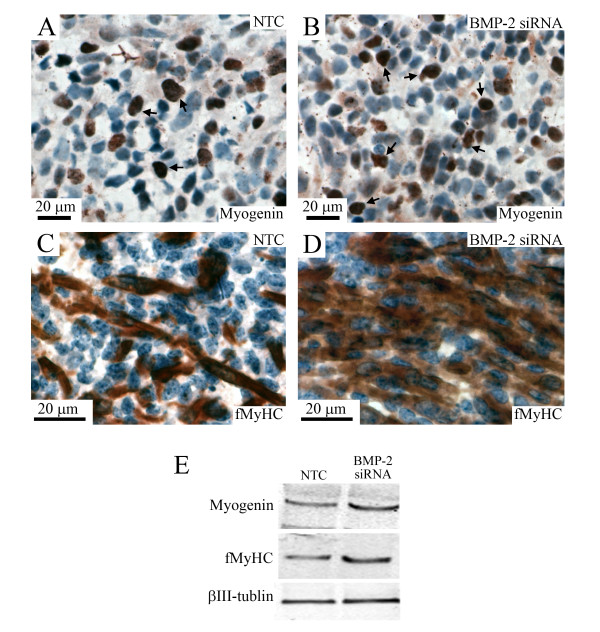
**BMP-2 siRNA induced the expressions of myogenin and fMyHC in cultured tongue**. Immunostaining images for myogenin (A, B) and fMyHC (C, D) in the middle portion of E13 tongues cultured for 8 days in BGJb medium containing 250 nM of the NTC (A, C) and BMP-2 siRNA (B, D). Brown color indicates immunostaining image for myogenin and fMyHC. Arrows in A and B indicate myogenin-positive nuclei. Western blotting pattern of myogenin, fMyHC, and βIII-tubulin in the whole portion of E13 tongues cultured for 8 days in BGJb medium containing the NTC and BMP-2 siRNA (E).

To further verify whether BMP-2 siRNA could stimulate the differentiation of myoblasts in cultured tongues, we analyzed the immunolocalization and expression levels of myogenin and fMyHC proteins in the NTC- and BMP-2 siRNA-treated tongues (Figure 5). The number of myogenin-positive cells appeared to be higher in the BMP-2 siRNA-treated tongue (arrows in Figure 5B) than in the NTC-treated tongue (arrows in Figure 5A). There were more elongated myotubes with multi-nuclei in the BMP-2 siRNA-treated tongues (Figure 5D) than in the NTC-treated tongue (Figure 5C). The expression levels of myogenin and fMyHC proteins were higher in the BMP-2 siRNA-treated tongue than in the NTC-treated tongue (Figure 5E).

### Suppression of BMP-2 expression by siRNA did not affect the formation of cartilage and bone in cultured tongue

To elucidate the effect of BMP-2 siRNA on the formation of cartilage and bone in cultured tongue, we analyzed the mRNA expression levels of Runx2, osteocalcin, ALP, collagen II, and collagen X, which are markers for chondrogenesis and osteogenesis (Table 2 and Additional file 1). Neither BMP-2 siRNA- nor NTC-treated tongue induced detectable levels of Runx2 mRNA. Further, there were no significant differences in the mRNA expression levels of osteocalcin, ALP, collagen II, and collagen X between the NTC- and BMP-2 siRNA-treated tongues. Their ratios relative to GAPDH were also extremely low, being approximately 1000-fold lower than those of the myogenic markers, and the variations for measurement were also large.

### Neither recombinant BMP-2 nor BMP-2 siRNA altered the expression of Id1

Since BMP-2 suppressed the differentiation of C2C12 myogenic cells by reducing the activity of the myoD family, such as myoD and myogenin, via up-regulation of Id1 [7-9], we analyzed the effects of recombinant BMP-2 and BMP-2 siRNA on the mRNA expression levels (Table 3 and Additional file 1). We found no marked difference in the mRNA expression levels of Id1 between the vehicle- and recombinant BMP-2-treated tongues, or between the NTC- and BMP-2 siRNA-treated tongues.

**Table 3:** mRNA levels of Id1

Vehicle- and BMP-2-treated tongues			
Genes	Vehicle	Recombinant BMP-2	Significance
Id1	0.000517 ± 0.000133	0.00054 ± 0.000184	NS

**NTC- and BMP-2 siRNA-treated tongues**			

	**NTC**	**BMP-2 siRNA**	

Id1	0.000454 ± 0.000264	0.000381 ± 0.000149	NS

The mean ± standard deviation of six vehicle-treated or six BMP-2-treated tongues for each gene is shown. The ratio of each target gene mRNA relative to GAPDH mRNA is shown.

## Discussion

In vitro studies using the myogenic cell line C2C12 demonstrate that BMP-2 converts the developmental pathway of C2C12 from a myogenic lineage to an osteoblastic lineage [7-11], whereas in vivo studies using null mutation mice demonstrate that BMPs inhibit the specification of the developmental fate of myogenic progenitor cells [12-17]. However, the roles of BMPs in the phases of differentiation and maturation in skeletal muscles have remained elusive. In the present study, recombinant BMP-2 suppressed the expressions of markers for the differentiation of skeletal muscle cells in cultured E13 tongue, whereas the suppression of BMP-2 with siRNA stimulated them, suggesting that BMP-2 functions as a negative regulator in the final differentiation of tongue myoblasts.

BMPs were first discovered as a factor that induced ectopic cartilage formation when implanted intramuscularly in adult rats [2,3]. However, in the present study, the formation of cartilage and bone was not observed in cultured tongue treated with recombinant BMP-2, and neither the recombinant BMP-2 nor BMP-2 siRNA altered the expression of markers for the formation of cartilage and bone, such as osteocalcin, ALP, collagen II, and collagen X. Collectively, these results suggest that BMP-2 functions as a negative regulator in the final differentiation of tongue myoblasts, but not as an inducer in the formation of cartilage and bone in cultured tongue.

In the present study, the transcriptional levels of chondrogenic and osteogenic markers were approximately 1000-fold lower than those of the myogenic markers, implying that the genes involved in myogenesis are in an activation mode, while the genes involved in chondrogenesis and osteogenesis are in a silencing mode. There may be several candidate mechanisms to regulate the status of gene expression. It was recently reported that epigenetic regulation, such as via the methylation and acetylation of histone, and the methylation of DNA, plays essential roles in the development and regeneration of several kinds of tissues [27,28]. Additionally, in the development of skeletal muscle, the transcription of myogenin, myoD, and MCK is reported to be regulated by the methylation or de-methylation of histone during myogenesis [29-31]. The silencing of genes involved in chondrogenesis and osteogenesis by epigenetic regulation is a good potential candidate mechanism suggesting that BMP-2 does not function as an inducer in the formation of cartilage and bone in cultured E13 tongue. To fully elucidate this issue, further studies will be necessary.

In vitro studies using myogenic cell line C2C12 demonstrate that BMP-2 converts the developmental pathway of C2C12 via an elevation in the expression of Id1 which suppresses the activity of the myoD family [7-11,32]. However, in the present study, the recombinant BMP-2 or BMP-2 siRNA did not induce an alteration in the expression of Id1, suggesting the existence of a different pathway(s) for the regulation of tongue striated muscle cells from C2C12 myogenic cells. The myoD family is known to play an essential role in the development of tongue muscle cells, but there is no report on the function of Id1 in these cells [23], and the molecular mechanism of the interaction between Id1 and the myoD family during the development of tongue muscle cells remains unknown.

We previously reported several different functions of peptide growth factors in the development of tongue muscle cells from limb and cultured myogenic cell lines: TGFα promotes early differentiation of mouse tongue myoblasts [33-35], whereas it does not affect the differentiation of the C2C12 myoblast [36]; and the signal of TGFβ3, but not of 1 or 2, plays a role in the early stages in the differentiation of mouse tongue muscle cells through TGFβ receptor I, TGFβ receptor II, and smad2/3 [37], but TGFβ1 inhibits the differentiation of limb skeletal muscle cells [38,39]. In addition to the function of growth factors, there are several studies showing other differences between tongue muscle cells, and limb and cultured myogenic cell lines, such that fMyHC is expressed not only in the myotubes and myofibers of tongue muscles but also in the myoblasts of tongue muscles, although it is not expressed in the myoblasts of trunk and limb muscles [40]. Our present result offers support for the theory that the program governing tongue myogenesis is more similar to those for limb and cultured myogenic cell lines than for head muscles, although it still differs from the former.

The coefficients of variance for the mRNA expression levels of genes related to myogenesis ranged from 9.1 to 38.9%, whereas those for genes related to osteogenesis and chondrogenesis ranged from 18.0 to 197.1%. The ratios relative to GAPDH of the osteogenic and chondrogenic marker mRNAs were extremely low, being approximately 1000-fold lower than those of the myogenic marker mRNAs. Since these values of the osteogenic and chondrogenic marker mRNAs appeared at near the detection limit for real-time polymerase chain reaction (PCR) analysis, the extreme low level of expression may reflect marked variation.

## Conclusions

In vitro studies using the myogenic cell line C2C12 demonstrate that BMP-2 converts the developmental pathway of C2C12 from a myogenic lineage to an osteoblastic lineage [7-11], whereas in vivo studies using null mutation mice demonstrate that BMPs inhibit the specification of the developmental fate of myogenic progenitor cells [12-17]. In the present study, BMP-2 functioned as a negative regulator for the final differentiation of tongue muscle cells, but not as an inducer for the formation of cartilage and bone in cultured E13 tongue, probably because the genes related to myogenesis are in an activation mode, while the genes related to chondrogenesis and osteogenesis are in a silencing mode.

## Methods

### Experimental animal

Pregnant ICR mice were purchased from Nippon Clea Co., Ltd. (Tokyo, Japan) and killed by cervical dislocation at E13. Embryos were isolated from uterine decidua and were removed from their membranes under a dissection microscope. Tongues for PCR analysis were removed, immediately frozen, and then stored at -80°C until use. Experimental protocols concerning animal handling were reviewed and approved by the Institutional Animal Care Committee of Tsurumi University School of Dental Medicine.

### Organ culture

Tongues of the E13 embryos were carefully microdissected and explanted. The explants were supported by membrane filters having a 0.8 μm pore size (type AABP, Millipore Corp., Bedford, MA, USA) on steel rafts and were cultured in BGJb medium (Life Technologies, Rockville, MD, USA). The cultures were maintained at 37°C in an atmosphere of 5% carbon dioxide and 95% air with medium changes every 2 days. Human recombinant BMP-2 (R&D Systems, Inc., Minneapolis, MN, USA) in the vehicle (4 mM HCl, 0.15% bovine serum albumin) or the vehicle only was supplemented to the culture BGJb medium at a final concentration of 4 μg/ml of human recombinant BMP-2. A cocktail comprising four kinds of BMP-2 siRNA (GAACACAGCUGGUCACAGAUU, GCAGCCAACUUGAAAUUUCUU, GCAAGAGACACCCUUUGUAUU, and CCACAGAGCUCAGCGCAAUUU) and NTC was purchased from GE Healthcare UK, Ltd. (Buckinghamshire, England). They were mixed with a cationic agent, oligofectamine (Invitrogen, Carlsbad, CA, USA), and were added to the culture BGJb medium at a final concentration of 250 nM. After 8 days in the culture, the explants were rapidly frozen and stored at -85°C until subsequent total RNA extraction, or immediately fixed in Bouin's or 4% paraformaldehyde solution for immunohistochemistry.

### RNA extraction, reverse transcription, and real-time PCR amplification

Total RNA extraction, reverse transcription (RT), and real-time PCR amplification were performed according to the manufacturer's specifications (Trizol, Life Technologies, Gaithersburg, MD, USA). The RNA was treated with 2 units of ribonuclease-free deoxyribonuclease I (Life Technologies, Gaithersburg, MD, USA), and was then reverse transcribed with 200 units of reverse transcriptase (SuperScript II, Life Technologies, Gaithersburg, MD, USA).

SYBR Green real-time PCR was performed on the Takara PCR Thermal Cycler Dice (Takara Bio, Inc., Shiga, Japan) using the following cycle parameters for all genes studied: denaturation at 95°C for 10 min, followed by 40 cycles of 95°C for 15 s for denaturation, and 55°C for 15 s for annealing and extension. The nucleotide sequences for the primers of all target genes are shown in Table 4. The mRNA quantity of each gene was calculated using a standard curve of the known concentrations of cDNA of each gene and were normalized by the quantity of GAPDH mRNA, a housing keeping gene. We disclosed the details of real-time PCR conditions in Additional file 2 following minimum information for publication of quantitative real-time PCR experiments (MIQE).

**Table 4:** Sequences of primer specific for target genes

Genes		
MyoD	Forward	5'-GAC GGC TCT CTC TGC TCC-3'
	Reverse	5'-AAG TGT GCG TGC TCC TCC-3'
Myogenin	Forward	5'-GAG CGC GAT CTC CGC TAC AGA GG-3'
	Reverse	5'-CTG GCT TGT GGC AGC CCA GG-3'
MCK	Forward	5'-CAA TAA GCT TCG CGA TAA GGA G-3'
	Reverse	5'-GAT GGG ATC AAA CAG GTC CTT G-3'
Id1	Forward	5'-ACG ACA TGA ACG GCT GCT ACT-3'
	Reverse	5'-GCT CAC TTT GCG GTT CTG G-3'
Runx2	Forward	5'-TTT AGG GCG CAT TCC TCA TC-3'
	Reverse	5'-TGT CCT TGT GGA TTA AAA GGA CTT G-3'
Osteocalcin	Forward	5'-TGC TTG TGA CGA GCT ATC AG-3'
	Reverse	5'-GAG GAC AGG GAG GAT CAA GT-3'
ALP	Forward	5'-CAC GGG CAC CAT GAA GGA AAA G-3'
	Reverse	5'-TGG CGC AGG GGC ACA GGA GAC T-3'
Collagen II	Forward	5'-ACT GGT AAG TGG GGC AAG AC-3'
	Reverse	5'-CCA CAC CAA ATT CCT GTT CA-3'
Collagen X	Forward	5'-TGG GTA GGC CTG TAT AAA GAA CGG-3'
	Reverse	5'-CAT GGG AGC CAC TAG GAA TCC TGA GA-3'
Cyclin D1	Forward	5'-AGG CGG ATG AGA ACA AGC AGA-3'
	Reverse	5'-CAG GCT TGA CTC CAG AAG GG-3'
CDK4	Forward	5'-ACG CCT GTG GTG GTT ACG CT-3'
	Reverse	5'-CCA TCT CTG GCA CCA CTG AC-3'
BMP-2	Forward	5'-GTG GTG GAA GTG GCC CAC TT-3'
	Reverse	5'-CTG TTT GTG TTT GGC TTG ACG-3'
BMP-4	Forward	5'-TCC TGG TAA CCG AAT GCT GAT-3'
	Reverse	5'-GCT GCT GAG GTT GAA GAG GAA-3'
GAPDH	Forward	5'-GAT GCT GGT GCT GAG TAT GTC G-3'
	Reverse	5'-GTG GTG CAG GAT GCA TTG CTG A-3'

### Histochemistry

Sagittal sections of cultured tongues were prepared at a 10 μm thickness with a cryostat. Immuno-enzyme stainings were performed as previously described [41,42]. To evaluate the differentiation of muscle cells in the tongue tissues, we performed immunolocalization for fMyHC and myogenin; and to evaluate the effect of BMP-2 siRNA, we performed immunolocalization for BMP-2 in the cultured E13 mouse tongue. Monoclonal antibody against fMyHC was purchased from Sigma-Aldrich, Inc. (St. Louis, MO, USA). Polyclonal antibodies against myogenin and BMP-2 were purchased from Santa Cruz Biotechnology, Inc. (Santa Cruz, CA, USA). In vivo tongue tissues at E14 were used for positive control of fMyHC and myogenin (Additional file 3). For control staining, the primary antibody was replaced with normal goat, rabbit, mouse IgG, or phosphate buffered saline (PBS). To detect bone and cartilage formation, Alizarin red or Alcian blue stainings were used, respectively.

### Western blot analysis

Cultured tongues were homogenized in 2% SDS, 62.5 mM Tris-HCl (pH 6.8), and 10% glycerol containing the cocktail of protease inhibitors. The homogenate was centrifuged at 8,000 rpm for 15 minutes and the supernatant was stored at -20°C until use. The protein concentration of the supernatant was measured by the BCM protein assay (Pierce, Rockford, IL, USA). After β-mercaptoethanol was added to the supernatant (final concentration, 5%), the supernatant was heated at 100°C for 5 minutes. The samples containing 25 μg total proteins were subjected to 5-20% gradient SDS-PAGE. After electrophoresis, the proteins were transferred onto a PVDF membrane (Hybond-P PVDF Membrane, Amersham Biosciences Corp., Piscataway, NJ, USA). The membranes were treated with Casein Solution (Vector Laboratories, Inc., Burlingame, CA, USA) for 3 hours at room temperature, and then incubated overnight at 4°C with primary antibodies. The following primary antibodies were used for Western blot analysis in the present study: mouse monoclonal antibodies against fMyHC (Sigma-Aldrich Inc., St. Louis, MO, USA) and βIII-tubulin (Promega Co., Madison, WI, USA), rabbit polyclonal antibody against myogenin, and goat polyclonal antibody against BMP-2 (Santa Cruz Biotechnology Inc., Santa Cruz, CA, USA). Immunoreactions were made visible using the Vectastain Elite ABC Kit and 3-amino-9-ethylcarbazole (AEC) (Vector Laboratories, Inc.). The banding images were captured using a digital camera (FinePix, Fujifilm Corp., Tokyo, Japan) and transferred to a personal computer. The intensity in the bands was measured by a Densitograph (ATTO Corp., Tokyo, Japan). βIII-tubulin was used as a loading control to confirm that the same amount of protein was loaded to each lane of SDS-PAGE.

### Statistical analysis

A Mann-Whitney U test was used to compare the median values between two groups.

## List of abbreviations used

ALP: alkaline phosphatase; BMPs: bone morphogenetic proteins; CDK: cyclin-dependent kinase; E: embryonic day; fMyHC: fast myosin heavy chain; GAPDH: glyceraldehyde-3-phosphate dehydrogenase; HGF: hepatocyte growth factor; Id: inhibitor of DNA binding/differentiation; IGF: insulin-like growth factor; MCK: muscle creatine kinase; MIQE: minimum information for publication of quantitative real-time PCR experiments; NTC: non-target control RNA; PBS: phosphate buffered saline; PCR: polymerase chain reaction; RT: reverse transcription; Runx: runt-related gene; TGF: transforming growth factor.

## Authors' contributions

KA carried out the culture, histology and PCR analysis. AY conceived of the study, participated in the design and coordination, and drafted the manuscript. TS and TF carried out histological analysis. ES participated in the culture and PCR analysis. YN participated in the design and coordination, and helped to draft the manuscript. All authors read and approved the final manuscript.

## Supplementary Material

Additional file 1 **Relative expressions of gene mRNAs related to myogenesis chondrogenesis, osteogenesis**. Relative expression levels of gene mRNAs related to myogenesis in E13 tongue cultured for 8 days in BGJb containing vehicle or 4 μg/ml of human recombinant BMP-2 (upper panel), and containing 250 nM of NTC or BMP-2 siRNA (lower panel). The longitudinal axis represents the percent value relative to the mean value of each target gene of vehicle- or NTC-treated tongue set at 100. Each column represents the mean of six cultured tongues. Click here for file

Additional file 2 **The details of real-time PCR conditions**. The details of real-time PCR conditions were provided following minimum information for publication of quantitative real-time PCR experiments (MIQE). Click here for file

Additional file 3 **Positive control images for fMyHC and myogenin**. Immunostaining images for fMyHC (A) and myogenin (B) in the middle portion of in vivo tongues dissected from mouse embryo at E14. Click here for file
