# Supplementary figures and images for: Bone morphogenetic protein-2 functions as a negative regulator in the differentiation of myoblasts, but not as an inducer for the formations of cartilage and bone in mouse embryonic tongue
Source: BMC Dev Biol. 2011 Jul 7;11:44. doi: 10.1186/1471-213X-11-44 (PMC3160908; doi:10.1186/1471-213X-11-44)

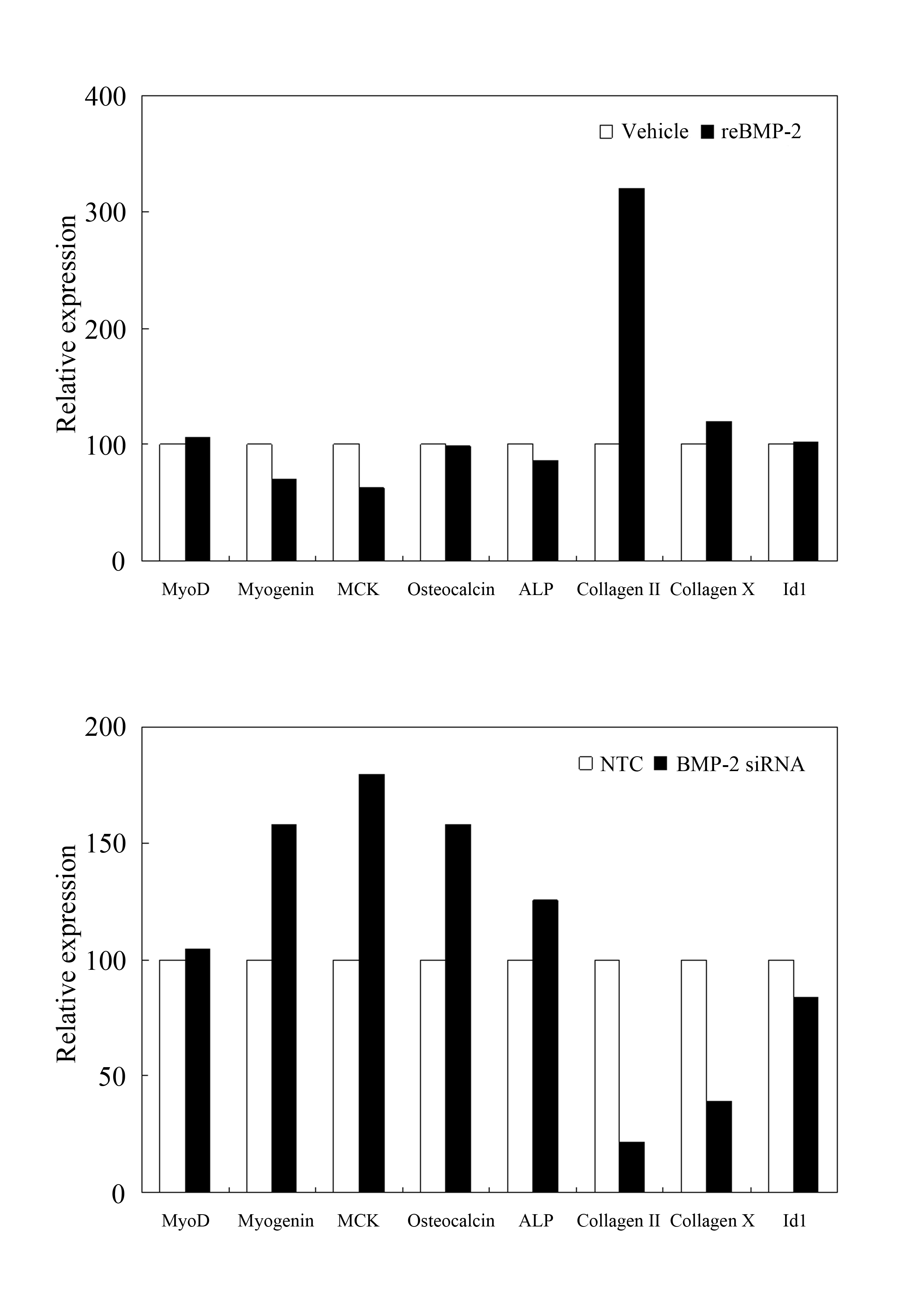

Supplement: Additional file 1 — Relative expressions of gene mRNAs related to myogenesis chondrogenesis, osteogenesis. Relative expression levels of gene mRNAs related to myogenesis in E13 tongue cultured for 8 days in BGJb containing vehicle or 4 μg/ml of human recombinant BMP-2 (upper panel), and containing 250 nM of NTC or BMP-2 siRNA (lower panel). The longitudinal axis represents the percent value relative to the mean value of each target gene of vehicle- or NTC-treated tongue set at 100. Each column represents the mean of six cultured tongues. [file 1471-213X-11-44-S1.TIFF]

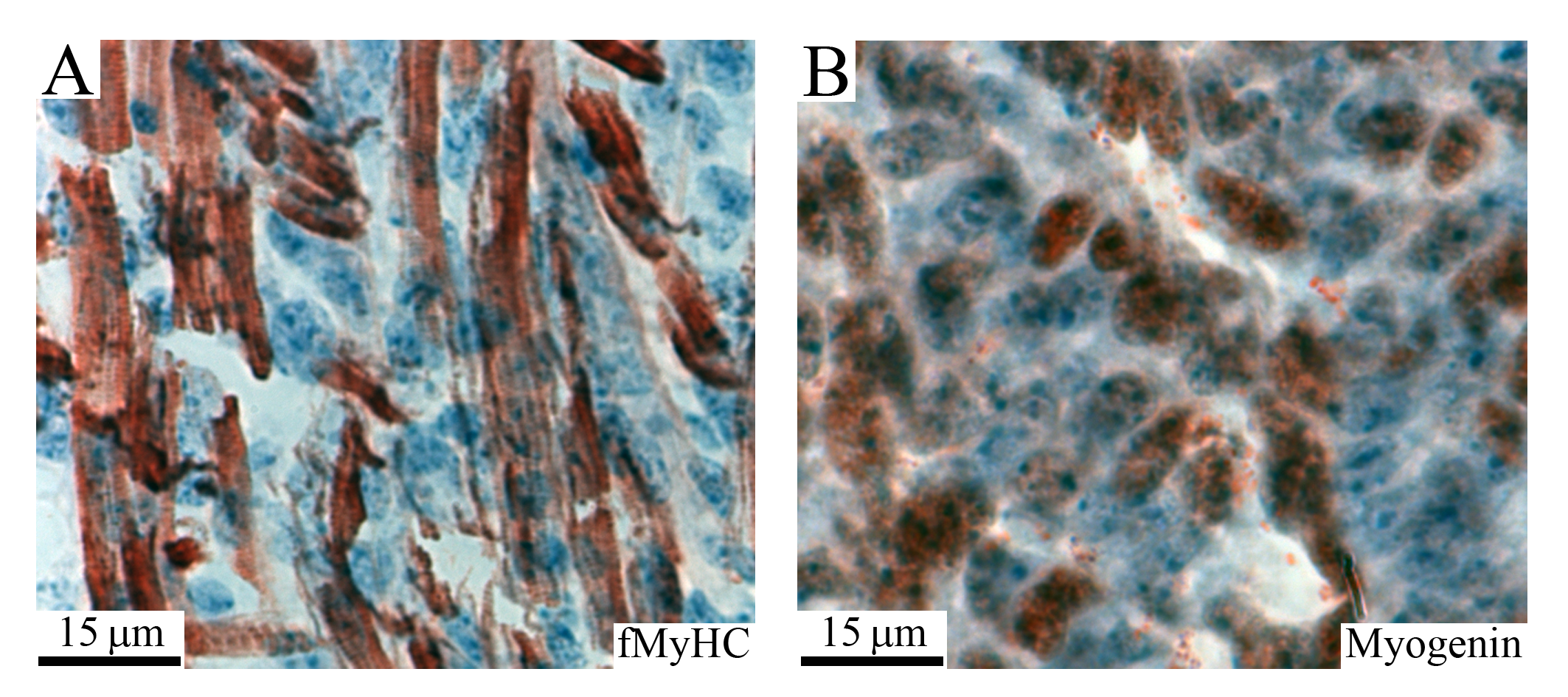

Supplement: Additional file 3 — Positive control images for fMyHC and myogenin. Immunostaining images for fMyHC (A) and myogenin (B) in the middle portion of in vivo tongues dissected from mouse embryo at E14. [file 1471-213X-11-44-S3.TIFF]
