# Supplementary material for: Bone morphogenetic protein-2 functions as a negative regulator in the differentiation of myoblasts, but not as an inducer for the formations of cartilage and bone in mouse embryonic tongue
Source: BMC Dev Biol. 2011 Jul 7;11:44. doi: 10.1186/1471-213X-11-44 (PMC3160908; doi:10.1186/1471-213X-11-44)
Supplement: Additional file 2 — The details of real-time PCR conditions. The details of real-time PCR conditions were provided following minimum information for publication of quantitative real-time PCR experiments (MIQE). [file 1471-213X-11-44-S2.DOC]

| **EXPERIMENTAL DESIGN** |
| --- |
| Definition of experimental and control groups |
| Number within each group |
| Assay carried out by core lab or investigator's lab? |
| Acknowledgement of authors' contributions |
| **SAMPLE** |
| Description |
| Volume/mass of sample processed |
| Microdissection or macrodissection |
| Processing procedure |
| If frozen - how and how quickly? |
| If fixed - with what, how quickly? |
| Sample storage conditions and duration (especially for FFPE samples) |

Definition of experimental and control groups was provided in the section of Methods. Number within each group was six cultured tongues. Assays were carried out in the investigator’s lab. Authors' contribution was described in the section of Authors’ contribution. Tongues were microdissected from E13 mouse embryo and cultured. One cultured tongue was used as one sample. After the culture, cultured tongues were quickly put in the -80°C freezer until use.

| **NUCLEIC ACID EXTRACTION** |
| --- |
| Procedure and/or instrumentation |
| Name of kit and details of any modifications |
| Source of additional reagents used |
| Details of DNase or RNAse treatment |
| Contamination assessment (DNA or RNA) |
| Nucleic acid quantification |
| Instrument and method |
| Purity (A260/A280) |
| Yield |
| RNA integrity method/instrument |
| RIN/RQI or Cq of 3' and 5' transcripts |
| Electrophoresis traces |
| Inhibition testing (Cq dilutions, spike or other) |

TRisol Reagent (Invitrogen, Cat#15596-018) was used for the RNA isolation, To remove any remaining DNA traces, RNA was treated with 2 unit of DNAse (Invitrogen, Cat#18068-015) in a 20 µl volume. All following procedures were performed according to the manufacturer’s instructions. Contamination was assessed by direct use of treated RNA in the qPCR reaction; additionally, since a melting curve (dissociation curve) is performed as standard, a contamination would be visible as additional peak. The precipitated RNA was resuspended in water. A 1:3 dilution was measured with the Ultraspec 2000 (Pharmacia Biotech). The A260/280 ratio is generally between 1.9 and 2.0, exact values for each RNA can be provided upon request. The yield is about 3 μg of RNA per a cultured tongue.

RNA integrity (RIN/RQI or Cq of 3' and 5' transcripts, Electrophoresis traces) and inhibition testing was not performed.

| **REVERSE TRANSCRIPTION** |
| --- |
| Complete reaction conditions |
| Amount of RNA and reaction volume |
| Priming oligonucleotide (if using GSP) and concentration |
| Reverse transcriptase and concentration |
| Temperature and time |
| Manufacturer of reagents and catalogue numbers |
| Cqs with and without RT |
| Storage conditions of cDNA |

Reverse transcription was performed in a 30 μl volume by 1.5 unit of SuperScript® III reverse transcriptase (Invitrogen, Cat#18080-085) for the generation of first strand cDNA. 1.5 µg of RNA (0.05 mg/ml), 0.7 µl of oligo(dT)_12-18_ (Cat#18418-012), 0.3 μl of random primer (Invitrogen, Cat#18427-088) and 1.5 µl dNTP mix (Invitrogen, Cat#18427-088) were incubated at 65°C for 5 min and quick chilled on ice. All other steps were performed by manufacturer’s instructions. For most cDNAs, the Cqs with RT are about 13 and without RT no amplification was detected. cDNA was stored in low adhesion tubes at -20°C.

| **qPCR TARGET INFORMATION** |
| --- |
| If multiplex, efficiency and LOD of each assay. |
| Sequence accession number |
| Location of amplicon |
| Amplicon length |
| *In silico* specificity screen (BLAST, etc) |
| Pseudogenes, retropseudogenes or other homologs? |
| Sequence alignment |
| Secondary structure analysis of amplicon |
| Location of each primer by exon or intron (if applicable) |
| What splice variants are targeted? |

Multiplex qPCR was not performed. Sequence accession numbers are X61655, X15784, X03233, U43884, DQ458792, X04142, X14174, M65161, X67348, S78355, L01640, L25602, X56848, X02231. Amplicon length is included in table under qPCR validation. *In silico* screen was performed with NCBI Blast and can be obtained from above web side. Primers were designed in exons. No splice variants were targeted.

| **qPCR OLIGONUCLEOTIDES** |
| --- |
| Primer sequences |
| RTPrimerDB Identification Number |
| Probe sequences |
| Location and identity of any modifications |
| Manufacturer of oligonucleotides |
| Purification method |

Primer sequences are included in the manuscript as Table 4. No modifications were used. Primers were purchase from FASMAC Co., Ltd. (Atsugi, Japan) and are salt-free.

| **qPCR PROTOCOL** |
| --- |
| Complete reaction conditions |
| Reaction volume and amount of cDNA/DNA |
| Primer, (probe), Mg++ and dNTP concentrations |
| Polymerase identity and concentration |
| Buffer/kit identity and manufacturer |
| Exact chemical constitution of the buffer |
| Additives (SYBR Green I, DMSO, etc.) |
| Manufacturer of plates/tubes and catalog number |
| Complete thermocycling parameters |
| Reaction setup (manual/robotic) |
| Manufacturer of qPCR instrument |

Each qPCR reaction had a 25 µl reaction volume containing:

cDNA corresponding to 50ng input RNA

400 nM of each forward and reverse primer

SYBR Premix Ex Taq (Takara, Cat#PR041A)

Tubes and lids were purchased from Takara Bio, Inc. (Cat#NJ300 and NJ302, respectively)

Cycling parameters were:

95°C for 10 min

95°C for 15 sec

55°C for 15 sec

Plate read

Cycle 40 times

Melting curve from 55°C to 95°C, read every 0.2°C, hold 2 sec

Reactions were set up manually in a designated room using designated equipment. qPCRs were performed with Takara PCR Thermal Cycler Dice (Takara Bio, Inc., Shiga, Japan).

| **qPCR VALIDATION** |
| --- |
| Evidence of optimisation (from gradients) |
| Specificity (gel, sequence, melt, or digest) |
| For SYBR Green I, Cq of the NTC |
| Standard curves with slope and y-intercept |
| PCR efficiency calculated from slope |
| Confidence interval for PCR efficiency or standard error |
| r2 of standard curve |
| Linear dynamic range |
| Cq variation at lower limit |
| Confidence intervals throughout range |
| Evidence for limit of detection |
| If multiplex, efficiency and LOD of each assay. |

The specificity of the amplification products have been confirmed by size estimations on a 3% agarose gel, sequencing of the products and by analyzing their melting curves. Without a template, no Cq could be determined since it never passed the threshold line.

| **Genes(Accession number)** | **length (bp)** | **slope** | **y-intercept** | **% efficiency** | **r2** |
| --- | --- | --- | --- | --- | --- |
| **MyoD(X61655)** | 146 | -3.50 | 21.35 | 99 | 0.9873 |
| **Myogenin(X15784)** | 379 | -4.70 | 25.41 | 98 | 0.9855 |
| **MCK(X03233)** | 160 | -3.01 | 21.24 | 99 | 0.9917 |
| **Id1(U43884)** | 71 | -2.93 | 17.60 | 99 | 0.9900 |
| **Runx2(DQ458792)** | 102 | -2.95 | 22.23 | 100 | 0.9900 |
| **Osteocalcin(X04142)** | 149 | -2.65 | 18.94 | 100 | 0.9981 |
| **ALP(X14174)** | 341 | -2.30 | 17.45 | 98 | 0.9782 |
| **Collagen II(M65161)** | 115 | -2.22 | 17.56 | 97 | 0.9738 |
| **Collagen X(X67348)** | 210 | -3.03 | 18.70 | 101 | 0.9988 |
| **Cyclin D1(S78355)** | 269 | -4.17 | 24.57 | 100 | 0.9974 |
| **CDK4(L01640)** | 280 | -2.25 | 16.83 | 95 | 0.9506 |
| **BMP-2(L25602)** | 186 | -3.90 | 22.55 | 97 | 0.9703 |
| **BMP-4(X56848)** | 436 | -3.85 | 21.18 | 98 | 0.9893 |
| **GAPDH(X02231)** | 197 | -3.31 | 22.78 | 100 | 0.9926 |

| **DATA ANALYSIS** |
| --- |
| qPCR analysis program (source, version) |
| Cq method determination |
| Outlier identification and disposition |
| Results of NTCs |
| Justification of number and choice of reference genes |
| Description of normalisation method |
| Number and concordance of biological replicates |
| Number and stage (RT or qPCR) of technical replicates |
| Repeatability (intra-assay variation) |
| Reproducibility (inter-assay variation, %CV) |
| Power analysis |
| Statistical methods for result significance |
| Software (source, version) |
| Cq or raw data submission using RDML |

qPCR analysis program (source, version): Takara PCR Thermal Cycler Dice System TP850 version 3.0

Cq’s were determined by setting the threshold to -1.0 using a log scale,

No data have been exclude from the calculations

Results of NTCs: no amplification products present thus no Cqs

Justification of number and choice of reference genes: cDNAs had previously been tested with

another reference gene (*S16*) with the same results

Description of normalisation method: GAPDH

Number and concordance of biological replicates: 3

Number and stage (RT or qPCR) of technical replicates: 3 at qPCR level, 2 for RT (Mn samples)

Repeatability (intra-assay variation): was below one Cq
